# Gap-induced inhibition of the post-auricular muscle response in humans and guinea pigs

**DOI:** 10.1016/j.heares.2019.01.009

**Published:** 2019-03-15

**Authors:** Caroline A. Wilson, Joel I. Berger, Jessica de Boer, Magdalena Sereda, Alan R. Palmer, Deborah A. Hall, Mark N. Wallace

**Affiliations:** a Medical Research Council Institute of Hearing Research, School of Medicine, University of Nottingham, Nottingham, UK; b Hearing Sciences, Division of Clinical Neuroscience, School of Medicine, University of Nottingham, Nottingham, UK; c National Institute for Health Research (NIHR) Nottingham Biomedical Research Centre, Ropewalk House, 113 The Ropewalk, Nottingham, UK; d Nottingham University Hospitals NHS Trust, Queens Medical Centre, Derby Road, Nottingham, NG7 2UH, UK; e University of Nottingham Malaysia, Jalan Broga, 43500, Semeniyh, Selangor Darul Ehsan, Malaysia

**Keywords:** Gap-pre-pulse inhibition, Preyer reflex, Acoustic startle reflex, Eye-blink reflex, Tinnitus, GPIAS, gap-induced inhibition of the acoustic startle, PAMR, post-auricular muscle response, PPI, pre-pulse inhibition

## Abstract

A common method for measuring changes in temporal processing sensitivity in both humans and animals makes use of GaP-induced Inhibition of the Acoustic Startle (GPIAS). It is also the basis of a common method for detecting tinnitus in rodents. However, the link to tinnitus has not been properly established because GPIAS has not yet been used to objectively demonstrate tinnitus in humans. In guinea pigs, the Preyer (ear flick) myogenic reflex is an established method for measuring the acoustic startle for the GPIAS test, while in humans, it is the eye-blink reflex. Yet, humans have a vestigial remnant of the Preyer reflex, which can be detected by measuring skin surface potentials associated with the Post-Auricular Muscle Response (PAMR). A similar electrical potential can be measured in guinea pigs and we aimed to show that the PAMR could be used to demonstrate GPIAS in both species.

In guinea pigs, we compare the GPIAS measured using the pinna movement of the Preyer reflex and the electrical potential of the PAMR to demonstrate that the two are at least equivalent. In humans, we establish for the first time that the PAMR provides a reliable way of measuring GPIAS that is a pure acoustic alternative to the multimodal eye-blink reflex. Further exploratory tests showed that while eye gaze position influenced the size of the PAMR response, it did not change the degree of GPIAS.

Our findings confirm that the PAMR is a sensitive method for measuring GPIAS and suggest that it may allow direct comparison of temporal processing between humans and animals and may provide a basis for an objective test of tinnitus.

## Highlights

• Myogenic potentials from the guinea pig pinna show gap induced pre-pulse inhibition.
• Startle inhibition is also shown by gaps in background noise using the Preyer reflex.
• Startle potentials recorded behind the human pinna show gap-induced inhibition.
• Human post-auricular muscle potentials may form an objective test for tinnitus.

## 1 Introduction

The main justification for undertaking animal neurophysiology is because it can give us an insight into how the human brain works in health and disease. Ideally any research should be cross-validated by using equivalent methods in animals and humans. This is particularly true in translational studies of clinical problems such as tinnitus (Eggermont, 2016). There are several objective tests used in animal models of tinnitus but the most popular involve modification of the acoustic startle response (Hayes et al., 2014; von der Behrens, 2014). The acoustic startle response involves many muscles including those in the limbs and the head. In small, active rodents it can be measured with a transducer in the cage floor as the animal “jumps” (Turner et al., 2006), while in larger, less active animals it can be measured by motion tracking cameras monitoring the ear flick or pinna reflex (Berger et al., 2013). However, in humans, these methods are not suitable and the eye-blink reflex is usually used instead (Fournier and Hebert, 2016). The lack of an equivalent test for animals and humans has two drawbacks. First, it has not been possible to confirm to what extent the animal behavioural methods assess the human perception of tinnitus. Second, one cannot be certain that the putative neural mechanisms for tinnitus that are derived from animal research are tinnitus-specific. They may equally be associated with other phenomena, such as hearing loss, insomnia or stress.

The most commonly used method for identifying tinnitus in animals is Gap-induced Inhibition of the Acoustic Startle (GPIAS); a form of pre-pulse inhibition (PPI). It relies on a short gap in a continuous background noise or tone to provide a cue that inhibits the usual startle response following a loud sound (Turner et al., 2006). The ratio between the magnitude of the response to the startling sound presented alone (no-gap trial) and trials in which a gap preceded the startling sound (gap trials) is calculated as the GPIAS ratio (Turner et al., 2006). If the gap is too short or if the tinnitus percept masks the gap then there will be no difference between the gap and no-gap trials.

The whole body reflex has been used to demonstrate tinnitus-related changes in GPIAS, in the mouse (Longenecker and Galazyuk, 2011; Moreno-Paublete et al., 2017), rat (Turner et al., 2006) and gerbil (Nowotny et al., 2011). However, one of the limitations of this method is that the whole body response habituates quite rapidly, especially in humans (Groves et al., 1974) where the eye-blink reflex is used instead (Fournier and Hebert, 2013; Shadwick and Sun, 2014). Although the eye-blink reflex has been used to demonstrate GPIAS, it has not yet been used to demonstrate the presence of tinnitus in humans and it is only rarely used in animals. Fournier and Hebert (2013) found that a group of tinnitus subjects did show a deficit in gap detection ability for the eye-blink reflex, but it was not specific for a background band-passed noise matched to the tinnitus pitch, as predicted in the original hypothesis (Turner et al., 2006). Psychoacoustic measures attempting to demonstrate a key assumption of the GPIAS method - that tinnitus “fills in” the gap in the background noise – have also failed (Campolo et al., 2013; Boyen et al., 2015). Thus it is still necessary to validate the GPIAS model in humans with tinnitus. Going forward it is worth noting that the whole body and eye-blink responses are not purely acoustic reflexes. They respond to startling stimuli presented in the visual or somatosensory domains (Yeomans et al., 2002). This will further complicate the interpretation of any results based on them.

**Fig. 1:**
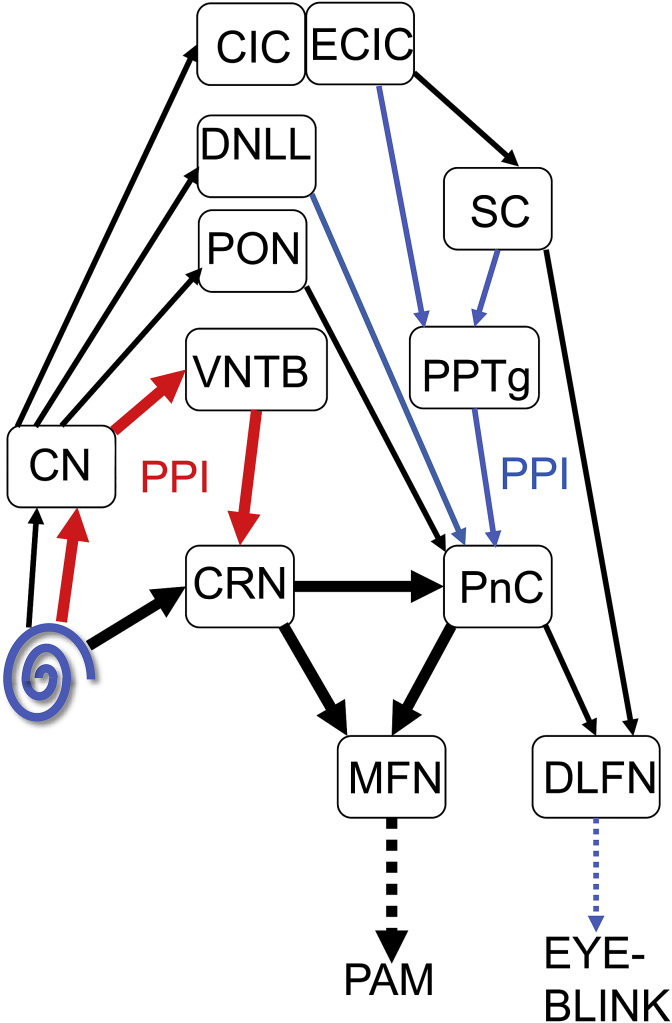
**Simplified diagram of the potential pathways involved in the mammalian acoustic startle reflex and its modification by pre-pulse inhibition (PPI).** There is a di-synaptic pathway from the cochlea to the cochlear root nucleus (CRN) and then to the medial facial nucleus (MFN) that innervates the posterior auricular muscle (PAM). There is also a tri-synaptic pathway from the CRN to the caudal pontine reticular formation (PnC) and then the facial nucleus. These are very short-latency pathways (thick black arrows). There is also a short-latency acoustic pathway which mediates PPI that is shown by the thick red arrows from the cochlea to the ventral cochlear nucleus (VCN), ventral nucleus of the trapezoid body (VNTB) and then to the CRN. Other pathways start at the cochlear nucleus and involve structures such as the periolivary nuclei (PON), dorsal nucleus of the lateral lemniscus (DNLL) and the central nucleus of the inferior colliculus (CIC). The CIC projects to the external nucleus of the inferior colliculus (ECIC) which has connections to both the superior colliculus (SC) and directly to the pedunculopontine tegmental nucleus (PPTg). The SC may project directly to the dorsolateral facial nucleus (DLFN) but the main input to the DLFN is from the PnC. Thus the PPTg may provide a longer latency route (shown by the thin blue arrows) for mediating multimodal PPI.

In guinea pigs, we have overcome the problem of habituation by measuring the Preyer or pinna reflex using infrared motion tracking (Berger et al., 2013, 2018). The Preyer reflex was first described in guinea pigs in the late 19th century (Preyer, 1882). It is a pure acoustic reflex, as it is not produced in response to startling stimuli in the visual or somatosensory domains (Fox et al., 1989; Hackley, 2015). In the rat, it involves a di-synaptic pathway (see Fig. 1) and this may also be true in the human (Hackley et al., 2017). Following a startling sound, large numbers of auditory nerve fibres simultaneously activate the cochlear root nucleus (CRN) which projects to the medial facial nucleus (MFN) which in turn innervates the muscles around the pinna (Horta-Junior et al., 2008) or ear such as the posterior auricular muscle (PAM). The CRN also projects to the caudal pontine reticular nucleus (PnC) which is involved in the whole body startle (Lee et al., 1996; Lingenhohl and Friauf, 1994) and this in turn projects to the MFN as well as the dorsolateral facial nucleus (DLFN) which innervates the orbicularis oculi muscle that is responsible for the eye-blink (Morcuende et al., 2002). The pathways mediating the acoustic startle reflex and their modulation by PPI are complicated and their details are not yet certain (Moreno-Paublete et al., 2017), but a simplified diagram summarising them is shown in Fig. 1. There is a short latency, purely acoustic pathway which mediates PPI that is shown by the thick red arrows (Gomez-Nieto et al., 2010). The broader acoustic startle response has multimodal inputs that can modify the general motor output and PPI involves many parallel pathways starting at the cochlear nucleus and feeding into the caudal pontine reticular nucleus as indicated by the blue arrows (Yeomans et al., 2006; Fendt et al., 2001; Koch et al., 1993; Li et al., 1998). The broader PPI measured by the eye-blink reflex is altered by certain psychiatric conditions such as schizophrenia and obsessive compulsive disorder (Kohl et al., 2013) and so studying modification of the pure acoustic reflex may be more appropriate for acoustic conditions such as temporal processing or tinnitus. The Preyer reflex shows robust PPI in rats (Cassella and Davis, 1986), as well as GPIAS in guinea pigs that can be used to identify tinnitus (Berger et al., 2013, 2018; Wu et al., 2016).

**Fig. 2:**
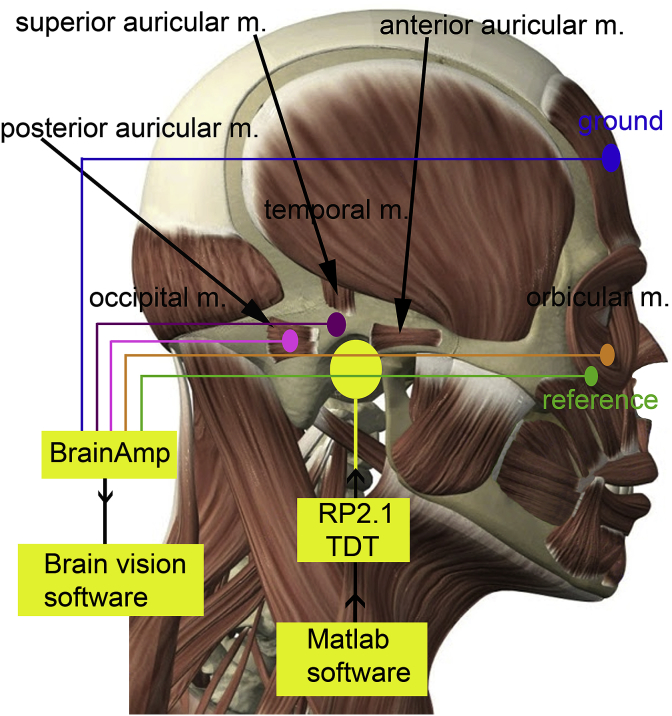
**Diagram of recording arrangement.** The active electrode (pink) was placed behind the right ear at the insertion of the muscle to the pinna, the PAMR reference (purple) and ground (blue) were placed at the tip of the pinna and centre of the forehead respectively. The eye-blink electrode (brown) was placed under the middle of the right eye and the eye-blink reference (green) was placed at the corner of the eye (approx. 1.5 cm apart). The diagram also illustrates stimulus production and electrode signal recording. TDT = Tucker Davis Technologies.

In humans, the Post-Auricular Muscle Response (PAMR) is a vestigial remnant of the Preyer reflex (Hackley, 2015). It can be measured non-invasively from a scalp electrode placed behind the ear, over the insertion of the muscle to the pinna (Fig. 2). One of the unusual characteristics of this muscle response is that it is amplified when the eye gaze is focused towards an extreme lateral position. Apparently this is due to the output from the superior colliculus that activates the oculomotor and abducens nuclei also innervating the MFN and increasing tonic activity in the PAM (Patuzzi and O'Beirne, 1999). Indeed, the PAMR reflex was originally described as being part of an oculo-auricular response (Wilson, 1908).

There are three muscles inserted into the base of the auricle (Gray, 1989; Smith and Takashima, 1980): the posterior, superior and anterior auricular muscles, as illustrated schematically in Fig. 2. The superior muscle may be partially covered by the temporal muscle, while the anterior muscle is generally smaller than the other two (Talmi et al., 1997). Thus, although all three muscles are innervated by the facial nerve, it is traditionally the posterior muscle that has been used for measuring the vestigial Preyer reflex (Dus and Wilson, 1975; O'Beirne and Patuzzi, 1999). In guinea pigs, the myogenic potential measured from immediately behind the pinna is also referred to as the PAMR for convenience.

This article evaluates the PAMR and GPIAS in guinea pigs and in young healthy human participants as an important precursor to translational research in tinnitus patients. Establishing the PAMR as a method for measuring GPIAS would also show its potential for studying auditory temporal processing more generally (Fournier and Hebert, 2016). The first section reports a study that directly compared the traditional Preyer reflex (pinna movement) to the PAMR, measured using a chronically implanted electrode in the same guinea pigs, in order to confirm that both can be used to demonstrate GPIAS. The second section deals with human volunteers and had four objectives: 1) directly compare the startle reflex measurements obtained from eye-blink recording to the PAMR in the same participants; 2) demonstrate proof-of-concept that the PAMR can be modified by preceding gaps in noise; 3) determine whether increasing the size of the PAMR potential by changing eye gaze position would make it easier to measure GPIAS; and 4) confirm whether there was an optimal gap position for producing GPIAS.

## 2 Comparing the GPIAS measured using the Preyer reflex and PAMR in guinea pigs

### 2.1 Materials and methods

***Animals*** All procedures were in accordance with the European Communities Council Directive (86/609/EEC) and were approved by the University of Nottingham Animal Welfare and Ethical Review Body. Experiments were conducted on a total of nine tricolour guinea pigs (two male, seven female) weighing between 440 and 750 g at the time of electrode implantation. Guinea pigs were group-housed on a 12:12 h light:dark cycle, and food and water were freely available.

***Recordings*** The flexion of the pinna, indicative of the Prefer reflex, was measured behaviourally using a motion-tracking system of three infrared cameras (Vicon Motion Systems, Oxford, UK). A reflective marker (4 mm diameter) was attached to each pinna using cyanoacrylate adhesive. The motion-tracking system used these markers to triangulate the position of the ears, and subsequently to track pinna movement during the presentation of startling stimuli. All data were analysed offline using Matlab (R2014b, MathWorks, MA, USA). Further details are given in Berger et al. (2013).

**Fig. 3:**
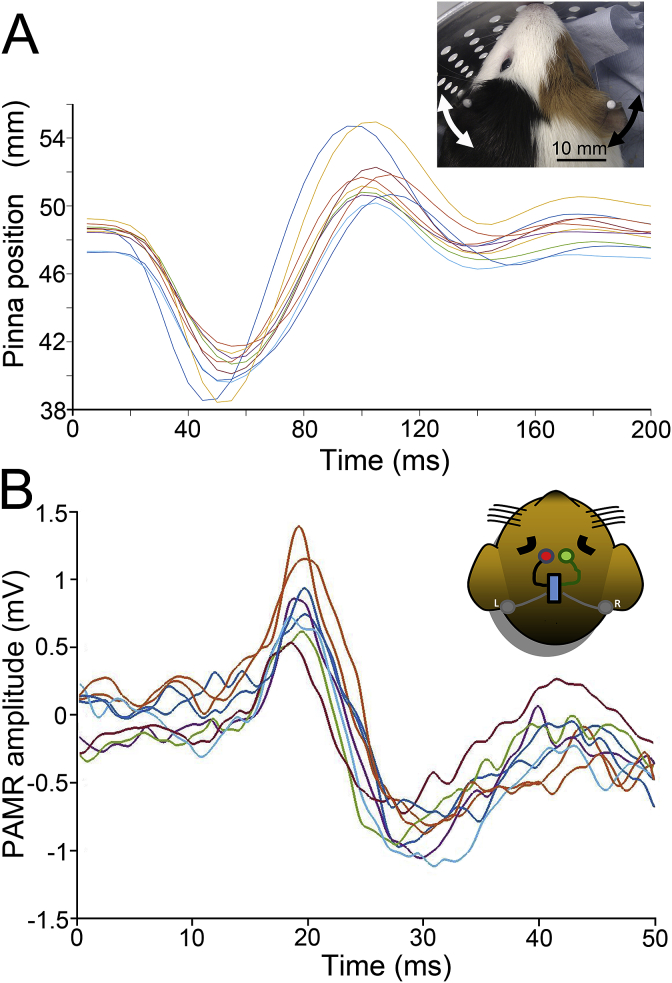
**Individual animal's raw Preyer reflex and PAMR traces, taken from #4.** Representative raw traces of no-gap trials (n = 10) overlaid for the Preyer reflex **(A)** and for the PAMR response (n = 8) **(B)** (with a single background condition of noise at 60 dB SPL, with a 100 dB SPL startle pulse). The inserts at the top right of each panel show the reflective markers which are tracked by the infrared cameras forming part of the Vicon system and the arrangement of the recording electrodes behind each pinna.

The PAMR was recorded using a chronically implanted electrode array. This comprised four Teflon-insulated silver wires, which were heated to produce a ball on the end to prevent them damaging the dura over the cortex. The wires were soldered to a circuit board attached to a Tucker Davis Technologies (TDT, Alachua, FL, USA) zero-insertion-force-clip connector. For the surgery, animals were anaesthetised with a mixture of ketamine (40 mg/kg, i.p. http://www.levetpharma.com/our-registrations/anaestamine-100-mgml-solution-for-injection/) and xylazine (8 mg/kg, i.p. www.drugs.com/vet/rompun-20-mg-ml-injectable-can.html) before being transferred to an isoflurane/O_2_ mixture from a face mask to maintain areflexia. Temperature was maintained at 38 ± 0.5 °C using a rectal probe and homeothermic blanket (https://www.harvardapparatus.co.uk/webapp/wcs/stores/servlet/haisku3_10001_11555_39108_-1_HAUK_ProductDetail_N_37610_37611_37613), the head was shaved and wiped with an iodine solution. Following a midline incision, the connective tissue from the top of the cranium was reflected and four burr holes drilled. Two of these were used to insert small, stainless steel anchoring screws and two were made over the frontal cortex so that ground and reference electrodes could be placed on the dura. The other two wires were pushed into a tunnel under the skin to lie on the muscle immediately behind the pinna (see Fig. 3B). The underside of the board and the electrode burr holes were covered in Kwik-Cast silicone rubber (https://www.wpi-europe.com/products/laboratory-supplies/adhesives/kwik-cast.aspx) and sealed in place with dental acrylic. The wound was sutured and the edges made to adhere to the acrylic using cyanoacrylate adhesive (https://www.3m.com/3M/en_US/company-us/all-3m-products/∼/3M-Vetbond-Tissue-Adhesive/?N=5002385+3294397973&rt=rud). All procedures were made using full aseptic precautions. Anaesthetic cream was applied to the wound, antibiotic (enrofloxacin) administered (https://www.baytril.com/en/farm-animals/product/) and the animal monitored until full recovery.

PAMR recordings were conducted at least 24 h later in a cage (310 × 150 × 210 mm) inside a sound-attenuated chamber, with a zero-insertion-force-clip headstage attached to the implanted electrodes. Animals were awake and freely moving throughout recording. Auditory stimuli were presented free-field via a single ¾-inch tweeter (http://www.mx-spk.com/image/XT19TD00-04-spec) positioned ∼30 cm above the centre of the cage. Two ¼-inch free-field microphones attached to a preamplifier (https://www.gras.dk/products/measurement-microphone-cartridge/externally-polarized-cartridges-200-v/product/645-40bp) and https://www.gras.dk/products/preamplifiers-for-microphone-cartridge/traditional-power-supply-lemo/product/675-26ac-1 placed at either end of the cage, were used to calibrate signals. Recorded EMG signals were filtered online between 60 and 300 Hz. Data was collected with a revised version of Brainware provided by its author (J. Schnupp, University of Oxford, UK).

***Stimuli*** Stimulus conditions for the Preyer reflex and PAMR were the same startling stimuli embedded in the same continuous background noise. The startle stimulus was a broadband (white) noise burst of 20 ms duration that included linear rise/fall times of 1 ms. There were five different continuous background noise conditions; four 2-kHz wide narrowband noise conditions centred at 5, 9, 13, and 17 kHz, and white noise, as described previously (Berger et al., 2018). Gaps of 50 ms duration, starting 100 ms before the startling stimulus, were randomly inserted on half of the trials, resulting in 10 ‘gap’/‘no-gap’ conditions for each background noise condition. Each animal separately underwent six Preyer reflex and six PAMR testing sessions on different days.

Sound presentation levels were determined individually for each animal prior to implantation, with startling stimuli of either 100, or 105 dB SPL and background carrier stimuli of 55, 60, or 70 dB SPL in a sound level-dependency test (Berger et al., 2013). The purpose was to avoid the startle sound being too loud for the response to be inhibited by a gap or being too soft so that the response rapidly habituates, as well as to optimise the test for each animal. At this point, one guinea pig (#9) was excluded because it failed to show any consistent evidence of GPIAS with the Preyer reflex.

***Data analysis*** Raw data for the Preyer reflex comprised x, y and z coordinates for each of the reflective markers captured by the Vicon motion-tracking software. Custom-written Matlab^©^ software was programmed to plot each individual startle response and calculate the peak-to-trough of the pinna displacement (see Berger et al., 2013).

For the PAMR collected in implanted animals, custom-written Matlab scripts (R2014b, MathWorks, MA, USA) were used for offline analysis. PAMR amplitudes were determined using peak-to-trough amplitudes of electromyographic potentials in the 50 ms following the startling stimulus, averaged across repeated trials.

For each animal and each background noise condition, the mean pinna displacement (mm) and mean PAMR amplitude (mV) for gap and no-gap trials were calculated. Both datasets were non-normally distributed and so a non-parametric Wilcoxon matched pairs signed rank test was performed to test the statistical significance (p < 0.05) between gap and no-gap trials. For illustrative purposes, the amount of GPIAS was also expressed as a percentage decrease in the pinna displacement/PAMR amplitude in gap trials compared to no-gap trials, which is equivalent to the GPIAS ratio used in other studies.

### 2.2 Results

#### 2.2.1 Comparing the GPIAS measured using the Preyer reflex and PAMR

Fig. 3 shows a representative set of raw traces of the Preyer reflex and PAMR for the no-gap startle trials. Table 1 reports the summary findings for all animals.

**Table 1:** **Comparison of GPIAS measured using the Preyer reflex and the PAMR**. Percentage GPIAS of the Preyer reflex and PAMR response for all guinea pigs for each background condition. The numbers in bold black represent statistically significant GPIAS values (p < 0.05). The numbers in grey indicate no significant GPIAS observed for that given background frequency. ID = individual participants, BBN = broadband noise.

ID	Preyer (GPIAS %)					PAMR (GPIAS %)				
	BBN	4-6 (kHz)	8-10 (kHz)	12-14 (kHz)	16-18 (kHz)	BBN	4-6 (kHz)	8-10 (kHz)	12-14 (kHz)	16-18 (kHz)
1	**46**	**22**	**36**	**32**	**29**	**15**	4	**22**	**33**	**13**
2	**36**	**23**	**17**	**17**	8	**49**	0	**24**	−9	**23**
3	**27**	**21**	**26**	**22**	**23**	**40**	**27**	**28**	**15**	**36**
4	**32**	**31**	**34**	**49**	**44**	2	**14**	**37**	**40**	**47**
5	**33**	**23**	**23**	**25**	**21**	**9**	**22**	**8**	**11**	**23**
6	**27**	**27**	**30**	**18**	**29**	**44**	**34**	**34**	**16**	**35**
7	**33**	**23**	**23**	**25**	**21**	−4	**13**	**17**	**18**	**31**
8	**41**	**18**	**35**	**22**	**21**	−16	**7**	−3	**6**	**25**
9	**51**	16	−3	**17**	−9	not done				

For the Preyer reflex, eight guinea pigs demonstrated statistically significant GPIAS in at least four of the five background noise conditions. The mean percent decrease in the pinna displacement in gap trials compared to no-gap trials was 27.3% (SD = 8.5). Comparatively speaking, for the PAMR the same eight guinea pigs demonstrated statistically significant GPIAS (p < 0.05) in at least three of the five background noise conditions. However, the mean percent decrease in the PAMR amplitude was somewhat reduced and more variable (mean = 19.8%, SD = 15.8). This difference in % GPIAS withstood statistical testing with a main effect of Preyer reflex versus PAMR (F _(4, 75)_ = 4.56, p = 0.036), but no main effect of background noise condition (F _(4, 75)_ = 0.97, p = 0.429).

Thus in our hands both the Preyer reflex and PAMR can be used to demonstrate GPIAS in the guinea pig. However, the Preyer response is larger and seems more robust.

## 3 Evaluating the PAMR in humans

### 3.1 Materials and methods

***Humans*** A total of 32 participants were recruited from around the campus by poster advertisements and word-of-mouth. All participants gave informed written consent and the studies were approved by the University of Nottingham, School of Medicine Ethics Committee (Ref: F11122014) on 5th January 2015. They were paid a small honorarium for the inconvenience of attending one or two sessions. Participants were aged 18–30 years with clinically normal hearing in both ears, normal (uncorrected) eyesight, and were fluent in English. After an otoscopic examination, hearing was assessed in each ear separately from 0.125 to 12 kHz using the British Society of Audiology (http://www.thebsa.org.uk/wp-content/uploads/2014/04/BSA_RP_PTA_FINAL_24Sept11_MinorAmend06Feb12.pdf) procedure with a Diagnostic Audiometer (GSI 16) in a sound proof booth. Normal hearing was defined by audiometric thresholds ≤20 dB Hearing Level in the frequency range 0.125–4 kHz.

Eight participants (four female, four male) were recruited in Study 1 which directly compared the conventional eye-blink reflex to the PAMR, in the same participants. One consented participant (male) was excluded from Study 1 due to thresholds ≥30 dB HL at 4 kHz. A further 24 different participants (18 female, six male) were recruited in Study 2 which examined the effect of various design parameters on the PAMR and corresponding GPIAS. Three of these were excluded because of elevated hearing thresholds and a further seven were excluded because they did not show a reliable PAMR response following 30 stimulus repetitions as defined in the Data Analysis section below. Eight completed study 2a (seven female, one male) which investigated the effect of eye gaze position on GPIAS and six completed study 2b (four female, two male) which investigated the effect of gap position on GPIAS.

***Stimulation*** Electrophysiological measurements took place in a sound-attenuating booth that also acted as a Faraday cage (IAC Acoustics, Winchester, UK). Participants were asked to sit quietly and refrain from moving their head. Eye gaze position was controlled by asking participants to fixate on a black cross that was placed on the facing wall. Short breaks were permitted between recording sessions in order to check on comfort and level of arousal. A recording session lasted approximately one hour and this included the attachment of electrodes and explanation of the procedure.

Stimuli were created using Matlab software (version r2014b, Mathworks, Natick, MA, USA). The startle stimulus was a 20-ms broadband noise burst presented at 105 dB SPL with near instantaneous rise-fall time (0.1 ms). No-gap trials consisted of startle pulses presented in a 1-kHz continuous background pure tone presented at 70 dB SPL. Gap trials were similar to no-gap trials, except that a silent gap (50 ms) was inserted in the continuous background tone before the startle pulse. In pilot studies, we had found that participants preferred a pure tone background rather than the white noise we have used in the guinea pigs. To reduce the risk of habituation of the PAMR response and to reduce anticipation of the startle stimulus, the inter-trial-interval (ITI) was randomly varied between 18 and 22 s. The gap duration was fixed at 50 ms and in the first study it started 100 ms before the onset of the startle as these parameters were used in previous eye-blink studies (Fournier and Hebert, 2013) and in our guinea pig work.

All stimuli were delivered to the right ear alone; in study 1 using circumaural Sennheiser HD-280 Pro headphones, and in study 2 using ER-1 inserts (https://www.etymotic.com/auditory-research/insert-earphones-for-research/er1.html). Transducers were connected to a Tucker Davis Technologies RP2.1 (Alachua, FL, USA) interface which was utilised as a digital signal processor and headphone amplifier (HB7).

Study 1 was a repeated-measures design with eye gaze position (0° “forward”, 30° “partially to the right”, 45° “fully right”) as the independent variable. The test session comprised of three testing blocks (one per gaze position starting at 0°) with each block containing 30 no-gap trials.

Study 2a was a repeated-measures design with eye gaze position (0° “forward”, 45° “fully right”) and gap/no-gap as the independent variables. The test session comprised of two testing blocks (one per eye gaze position) with each block containing a random sequence of 60 gap and 60 no-gap trials. Study 2b, was a repeated-measures design with gap condition (gap, no gap) and gap position (20, 50, 100 and 500 ms) as independent variables. The values of gap position reflected the interval between the end of the gap and the start of the startle stimulus. Eye gaze position was fixed throughout in the forward position. There were four testing blocks (one per gap position), with each block containing a random sequence of 20 gap and 20 no-gap trials and the blocks presented in a randomised order. Gap duration was fixed at 50 ms across both studies.

***Recording procedures*** Eye-blink reflex and PAMR were recorded at a sampling rate of 2500 Hz and filters set at 0.1–250 Hz using a BrainAmp DC system (BrainVision, Gilching, Germany) with 10 mm cupped AgCl electrodes fitted with impedances below 3 kΩ. PAMR electrode placement was guided by methods in Patuzzi and O'Beirne (1999). For the PAMR, the active electrode was placed behind the right (ipsilateral) ear, over the insertion of the muscle to the pinna (Fig. 2), with the reference electrode on the tip of the pinna (to avoid any intrinsic muscle responses) and the ground electrode on the centre of the forehead (Benning et al., 2004). For the eye-blink reflex, the active electrode was placed under the middle of the right (ipsilateral) eye, with the reference electrode at the corner of the eye at a distance of about 1.5 cm (Blumenthal et al., 2005).

***Data analysis*** All data was analysed using custom-made Matlab software (version r2014b) with EEGLAB toolbox (SCCN, University of California, San Diego, USA). The data were rectified and filtered offline using a bandpass filter of 1–300 Hz (Patuzzi and O'Beirne, 1999) to exclude neurogenic potentials (Thornton, 1975). For detecting a reliable response, a criterion threshold was defined as 2.5 times the standard deviation of the mean of the baseline, and the baseline was defined as a 2-s segment of the signal prior to the acoustic startle.

**Fig. 4:**
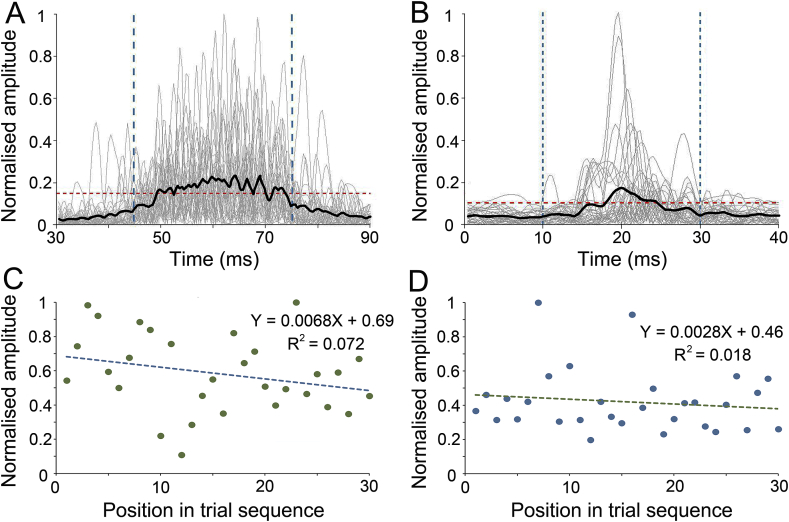
**Individual participant's eye-blink and PAMR traces, taken from #4.** Individual trials are shown by the grey lines for eye-blink (A) and PAMR (B). The black line represents the averaged waveform. The blue dashed lines indicate the respective windows of analysis. The red dashed line represents the criterion threshold for detecting a reliable response. Normalised amplitudes of the eye-blink (C) and PAMR (D) are displayed across the testing session. The dashed lines represent the trend over time and the slope and regression coefficient are shown for each line.

As the peaks in the individual traces differed in latency (Fig. 4), a window of analysis was specified. For the eye-blink reflex, this was 45–75 ms and for the PAMR it was 10–30 ms (Fournier and Hebert, 2013; Patuzzi and O'Beirne, 1999). Additionally, due to the differences in the mean amplitude between participants, each data set was normalised whenever data from different subjects were to be compared directly. Normalised individual PAMR responses were obtained by taking each data point and dividing by the largest data point value in all of the session data. For each participant who exhibited a reliable PAMR, the percentage GPIAS of the PAMR was calculated using a ratio of the peak-to-baseline measure of the amplitudes for gap and no-gap trials, using the formula: 100-(mean PAMR amplitude gap trials/mean PAMR amplitude no-gap trials)*100. As in the guinea pig data, mean PAMR amplitudes (mV) were non-normally distributed and so non-parametric Wilcoxon matched pairs signed rank tests were performed.

### 3.2 Results

#### 3.2.1 Comparing the eye-blink response and PAMR responses

In the initial recordings comparing the two reflexes (first part of study 1) the simplest set of conditions was used where the eyes were in the forward position and the startle was presented without any preceding gap. A representative set of raw traces of the eye-blink reflex and the PAMR are shown for an individual participant in Fig. 4. In both cases, the mean response waveform bore little resemblance to that of the individual trials. For individual trials, the eye-blink response was usually characterised by multiple peaks, whereas the PAMR typically had a single peak. Across trials, individual eye-blink responses varied in their maximum peak latency (49–75 ms) to a greater degree than the PAMR (14–26 ms).

The amplitude of the maximum peaks for the eye-blink and PAMR varied over their respective recording sessions (Fig. 4C and D). When the amplitude for each trial was plotted across the session, there was a weak trend towards declining amplitudes over time, with the slope of the regression line for the eye-blink response more than twice as steep as that for the PAMR. However the variability in amplitudes were so variable that overall there was no statistically significant linear reduction for either type of recording.

**Fig. 5:**
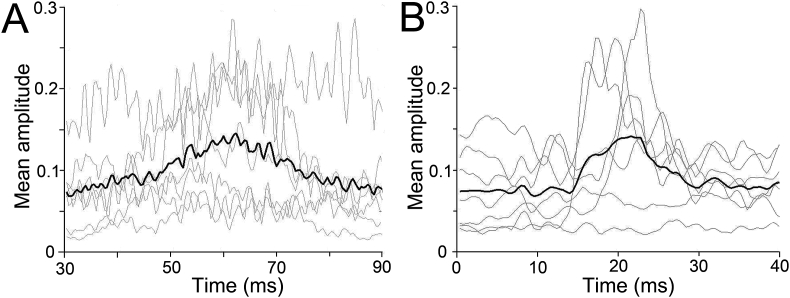
**Average eye-blink responses and PAMR, for all seven participants.** Mean traces for individual subjects are shown by the grey lines for eye-blink (A) and PAMR (B). The black line represents the group averaged waveform.

Out of seven participants only two showed mean eye-blink responses above threshold, whereas four showed mean PAMR responses above threshold. When averaged across the group, the magnitude of the amplitude was comparable for both types of recordings, but the average PAMR response was more clearly defined than the average eye-blink response, and it had a single primary peak and a narrower range of latencies. This is illustrated in Fig. 5.

#### 3.2.2 Processing optimisation to detect the PAMR response to allow comparison of the gap and no-gap conditions

**Fig. 6:**
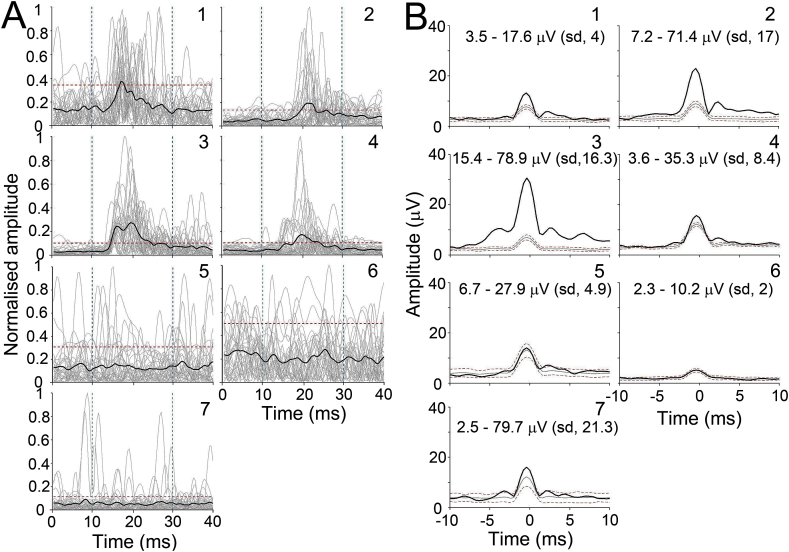
**Comparison of mean raw traces with the average aligned traces for the PAMR. A** Mean normalised responses from all seven participants showing the average waveform (black) in relation to the threshold (red dotted line) with the eyes straight ahead. **B** Average waveform of aligned PAMR traces in individual participants (black). The average waveform for the baseline, taken from outside the acquisition window, is represented in grey with the red dotted lines showing values at ± 2.5 times the standard deviation of the baseline, plus the mean of the baseline. The range of actual values for each participant is shown at the top of each panel along with the standard deviation (sd).

Next, a different form of analysis was used to more appropriately reflect the shape of the underlying potential in each trial and give a more accurate indication of whether or not a PAMR response could be detected from an average of the first 30 trials. This was based on a method for aligning peaks that has previously been used for analysing visual evoked potentials (McGillem and Aunon, 1977). The fact that the PAMR response was typically a single peak with a relatively narrow range of latencies meant that it was possible to produce a group-averaged waveform that contained little smearing caused by latency shifts from trial to trial. To achieve this, the highest value of the predominant peak in each trial was set as the zero timepoint and the adjacent segment of trace (±10 ms) was aligned, for all 30 trials, so that an adjusted waveform was obtained for each participant. This was then compared to the average aligned waveform of the greatest peak from a previous 2 s of baseline trace, starting at 3 s before the startle pulse. The individual averaged responses using the unaligned data for all seven participants is shown in Fig. 6A, where only the four participants (labelled #1–4) gave a response that was above threshold. The corresponding responses generated by the alignment procedure are shown in Fig. 6B. The adjusted waveforms were sharper and greater in amplitude. As a result, the data for participant #7 now reached the threshold for defining a significant response. In Fig. 6B, the mean of the highest peak outside the acquisition window, within a 2 s segment of baseline, is plotted as a grey line and the red dotted lines show values for ±2.5 times the standard deviation of these peaks across trials. This method of alignment appeared to provide a more sensitive way of estimating the PAMR than a conventional stimulus-linked average, and the amplitude of the adjusted waveform appears to be a more appropriate way of estimating response magnitude when comparisons between different conditions are needed.

**Fig. 7:**
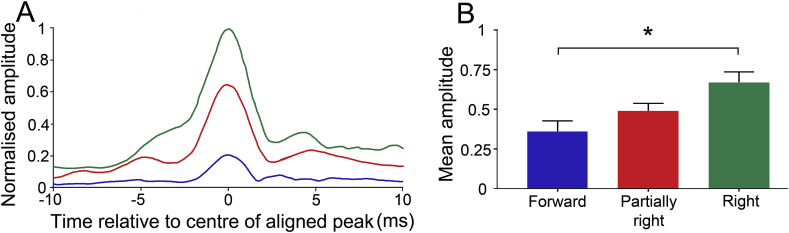
**Effect of eye direction on PAMR amplitude. A** Average aligned waveforms for participant #2 for each eye-gaze position (“forward” - blue, “partially right” – red, and “fully right” - green). **B** The bar chart shows the mean amplitude of the peaks taken from the five participants with a detectable PAMR response for the three eye-gaze positions. Dunn's test was used to show a significant difference between the forward and fully right eye-gaze conditions (*p < 0.05).

In the second part of study 1 we wanted to confirm the optimal eye gaze position to maximise the amplitude of the PAMR. Our research question focussed on whether eye gaze position affected the PAMR response and so did not evaluate the effect of eye position on the eye-blink data in this or subsequent experiments. Those five participants with a detectable PAMR response when the eye gaze was directed forward were tested with the additional conditions of eye gaze partially right and eye gaze fully right. An example of the aligned averaged PAMR waveform with the eye gaze at all three positions is shown in Fig. 7A (participant #2). Results showed that the aligned averaged PAMR waveform progressively increased in amplitude as the eye gaze became more lateralised. This pattern was true for all five participants and the grand average from the five participants is shown in Fig. 7B. A non-parametric Friedman test was used to test differences between the three eye gaze positions. The result of this test showed that the amplitude was dependent on the eye gaze position; χ^2^ (2, N = 5) = 8.4, p = 0.0085. Post-hoc analysis with a Dunn's test demonstrated a significantly greater amplitude in the fully right condition compared to the forward condition (p = 0.013). There was no significant difference between the forward and partially right, or between the partially right and fully right conditions (p > 0.999 and p = 0.1733, respectively).

#### 3.2.3 Gap induced inhibition of the PAMR response

**Fig. 8:**
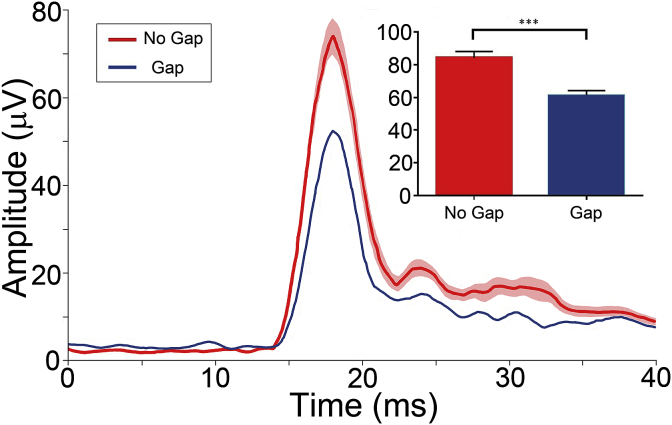
**Representative example from a single participant demonstrating GPIAS.** Average aligned waveforms for no-gap (red) and gap (blue) conditions (mean of 60 trials; ± Standard Error (SE) in pink for the no-gap trials. The SE for the gap trials was too small to plot). **Inset:** Histogram showing the mean peak-to-baseline amplitude of the PAMR in response to no-gap and gap conditions with a 27% reduction in PAMR amplitude following the gap (***p < 0.001).

Having optimised the method for estimating the PAMR response, the next step was to test whether it was possible to reduce PAMR amplitude by preceding it with a gap in a continuous sound (GPIAS). In this part of the study, the peak responses were aligned across trials and we also studied the effect of eye gaze position (Group 2a, n = 8) and gap position (Group 2b, n = 6) on the efficacy of the gap in reducing the PAMR response to the subsequent startle pulse. An example of GPIAS in an individual participant with eye gaze directed forward is shown in Fig. 8. In this participant, the adjusted PAMR response significantly decreased in amplitude when the gap condition was compared to the no-gap condition, demonstrating a reduction of 27% (Wilcoxon rank-sum test; p < 0.001).

**Fig. 9:**
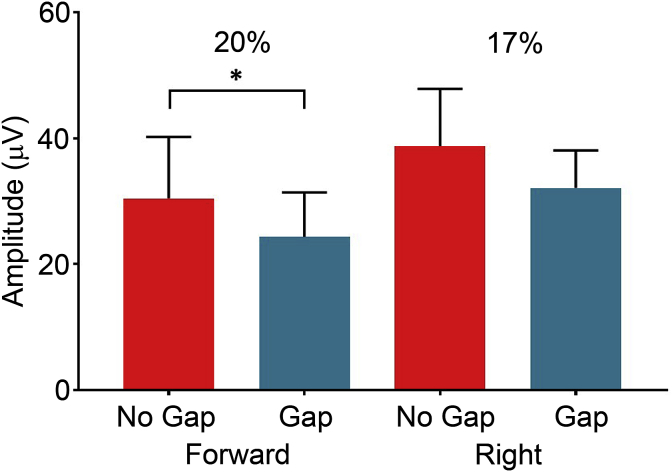
**Average GPIAS values for different eye-gaze positions.** Mean GPIAS scores from 8 participants of gap and no-gap trials for each eye-gaze condition. The forward condition displayed a 20% reduction (p = 0.031) and the right condition illustrated a 17% reduction (p = 0.156).

The data were then analysed for all eight participants in group 2a to determine if a more reliable GPIAS could be obtained with the eyes gazing right-ward compared to forward and the results are summarised in Fig. 9. The two eye gaze positions demonstrated similar GPIAS reductions; right-ward 17% and forward 20%. The Wilcoxon matched pairs signed rank test demonstrated a significant reduction across the no-gap and gap conditions (Z = −26, p = 0.031) for the forward eye gaze position, but not for the right-ward position (Z = −18, p = 0.156). A paired *t*-test was then used to determine if there was a significant difference in the amount of GPIAS that was obtained in the two eye gaze positions, but no difference was found (t(6) = 1.162 p = 0.289).

In conclusion, although the right-ward position increased the amplitude of the PAMR response, it did not increase the degree of GPIAS that could be demonstrated. As the forward eye gaze position showed a more reliable GPIAS and was more tolerable for participants than the right-ward conditions, the forward position alone was used in subsequent testing.

**Fig. 10:**
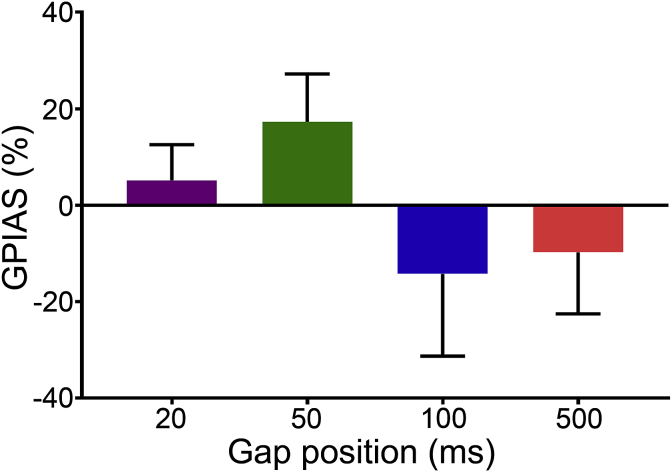
**Average GPIAS inhibition values for different gap positions.** Mean GPIAS % from six participants for each gap position. Both the 20 ms and 50 ms conditions demonstrated GPIAS of 5 and 17% respectively. The 100 ms and 500 ms conditions illustrated gap induced facilitation of the startle response.

In group 2b, six participants were tested with stimuli where the 50-ms gap was placed at different times before the startle pulse to examine the delay between the end of the gap and the start of the pulse. Four delays were used and the results summarised in Fig. 10. The 20- and 50-ms gap conditions showed a mean GPIAS value of 5% and 17% respectively, while the 100- and 500-ms conditions exhibited a gap induced facilitation value of 14% and 10%, respectively. A repeated-measures one way ANOVA with a Greenhouse-Geisser correction determined that GPIAS values obtained for each gap position did not differ significantly from one another (F(1.214, 0.753) = 0.561, p = 0.729). Although the variance in the data was rather high, the observed pattern suggested that the 50-ms gap position might be optimal for demonstrating GPIAS.

## 4 Discussion

### 4.1 Use of the PAMR as a measure of the acoustic startle response

When recordings are made from the human scalp, as many as 15 separate potentials can be identified following a brief acoustic pulse (Picton et al., 1974). The cranial muscular responses show temporal overlap and it can be difficult to disentangle them to identify a single source (Streletz et al., 1977). The post-auricular muscle usually has a single belly that is small and well-defined (Talmi et al., 1997) and contains a relatively small number of muscle units which are spontaneously active (De Grandis and Santoni, 1980). The acoustic startle synchronises the muscle unit activity to give a short latency potential that can be measured from the skin surface behind the ear. By placing a reference on the ear lobe, it is possible to obtain a relatively pure signal without much interference from other cranial muscles. The post-auricular muscle does not usually produce any measurable movement of the auricle, but its activation does seem to be analogous to the ear flick reflex shown by many mammals (Hackley, 2015). This view is supported by our results showing that the electromyographic response measured in the post-auricular area of the guinea pig is a short-latency potential that can be used to demonstrate GPIAS in the same way as the ear flick (Preyer) reflex. The PAMR has a low threshold for activation and should be present in participants with moderate hearing loss (Thornton, 1975; Yoshie and Okudaira, 1969). Its main advantage is that it shows almost no sign of habituation, even after thousands of repeats (Hackley et al., 1987) and it is becoming more widely used in psychology for measuring behaviour such as appetitive responding and PPI (Hackley et al., 2017; Sandt et al., 2009).

### 4.2 Comparison of the eye-blink response and PAMR for measuring changes in the acoustic startle

In the first part of the study we wanted to directly compare the eye-blink response and PAMR traces as alternative ways of measuring the acoustic startle reflex in a way that might be relevant in the clinic. Both the eye-blink and the PAMR responses are modulated by the emotional state of the participant, with aversive states, such as fear, potentiating the eye-blink and suppressing the PAMR while pleasant or appetitive states potentiate the PAMR and suppress the-eye blink (Benning et al., 2004; Vrana et al., 1988). A few participants found it unpleasant to have to maintain their eye gaze in a fixed side-ward position and two started to feel nauseous towards the end of a trial. Thus, we wanted to keep the test periods to a minimum and use recording sessions that could be completed in less than an hour where there was less chance of an aversive state building up than with a longer session. We never used more than 30 repeats in one continuous test block and this is less than would normally be used for recording the PAMR (O'Beirne and Patuzzi, 1999). Despite this data reduction, we were still able to detect a PAMR in 68% (19/28) of our participants and we found it easier using the PAMR than the eye-blink response to record a response that is suitable for averaging across trials. The raw PAMR trace generally had a single prominent peak which produced a smooth clear potential when these peaks were aligned and averaged across trials. We recommend that this adjusted waveform is better suited for directly comparing the response amplitude across gap and no-gap conditions. By contrast, the raw eye-blink trace was composed of multiple myogenic potential peaks. This meant that aligning one peak from each trace for averaging could misidentify valid responses and include them in the background activity, thus increasing the standard deviation of the background. Even after appropriate filtering to smooth the trace, the resultant averaged waveform peak would be broader and potentially have a lower signal-to-noise ratio than the PAMR peak recorded under the same conditions. This may make the eye-blink response sub-optimally sensitive to small changes in the peak amplitude produced by gap-induced inhibition. Thus, if the GPIAS led to a smaller number of muscle units being activated this might lead to a sharpening of the averaged response rather than a significant reduction in amplitude. By aligning the traces according to the largest peak in each trial, there might not be any reduction in amplitude until some of the trials had no motor units responding at all.

In our hands, 68% of participants showed a measurable PAMR and this is a bit lower than the 80% or more of participants that have been shown to have a PAMR in previous studies (O'Beirne and Patuzzi, 1999; Sandt et al., 2009) which generally used larger numbers of repeats (100 trials or more). Previous studies also showed that the background electromyographic activity in the post-auricular muscle could be potentiated by increasing the activity in other cranial muscle groups. Thus flexing the neck or smiling can increase the tonic activity and increase the amplitude of the PAMR (Dus and Wilson, 1975). Similarly activation of the oculomotor units involved in moving and holding eye gaze towards the side of the acoustic stimulus has been shown to increase the amplitude of the PAMR (Patuzzi and O'Beirne, 1999). We confirmed that finding, but were unable to show that the larger PAMR response was associated with a larger percentage GPIAS. This may just mean that the inhibition is a proportional effect. In other words, it does not matter what is the absolute amplitude, the magnitude of the change is a constant proportion.

### 4.3 Validation of the GPIAS method as an objective test for tinnitus in animals

In the original description of the GPIAS method for detecting tinnitus in rats (Turner et al., 2006), it was suggested that tinnitus acts to fill the gap in the background noise when its pitch and approximate bandwidth has been matched with the tinnitus percept. However, psychoacoustic attempts to confirm this mechanism using human subjects have been unsuccessful (Boyen et al., 2015; Campolo et al., 2013). When the tinnitus pitch was matched to the background noise in humans, there was no evidence of a greater effect on GPIAS of narrowband noise matched to the tinnitus pitch compared to noise centred at a well-separated pitch (Fournier and Hebert, 2013). Furthermore, direct measures of conscious gap detection in tinnitus patients failed to show any deficits that would significantly affect the 50-ms gap typically used in demonstrating GPIAS (Fournier and Hebert, 2016), although, as we have previously indicated, there are likely fundamental differences between gap-induced reductions of a reflex response and absolute gap detection thresholds (Berger et al., 2017).

Despite the lack of support from current human studies, the GPIAS test does give results that are consistent with the presence of tinnitus in many animal studies (Galazyuk and Hebert, 2015; Turner and Larsen, 2016). This implies that tinnitus may be affecting the unconscious neural processing of GPIAS in the brainstem rather than through altering conscious gap detection. The effect seems to be specific for the gap, as the effect of a brief noise pre-pulse, in reducing the response to a startle pulse, is not changed in animals where tinnitus has been induced (Dehmel et al., 2012). In both cases an alteration in the gain control of the output from the cochlear nucleus might be enough to change the strength of GPIAS. However, to validate the GPIAS method for use in animals it will be necessary to demonstrate a reduced level of GPIAS in tinnitus patients compared with an age and hearing loss matched control population. Until this is done the link between GPIAS and tinnitus will remain uncertain.

### 4.4 Development of an objective test for tinnitus in humans

One of the challenges for tinnitus research is that, even though there is great variety in the methods used for identifying tinnitus in animals, all are fundamentally different from the mainly questionnaire-based methods of the clinic. Human studies of tinnitus have involved measuring spontaneous oscillations in the cortical EEG activity (Adjamian, 2014) and more recently cortical evoked potentials (Han et al., 2017), but these have been of limited usefulness because it has not been practical to measure the activity in a single subject before and after the onset of tinnitus. The GPIAS method has been used in humans, in an attempt to detect tinnitus, by measuring the eye-blink reflex as a component of the general startle response (Fournier and Hebert, 2013; Shadwick and Sun, 2014). Although there were deficits in gap detection ability, they were not specific for a background noise matched to the tinnitus frequency. Furthermore, it is difficult to measure the electromyographic response associated with the eye-blink in awake animals where it would be possible to induce tinnitus experimentally (Servatius, 2000).

It is thought that the PAMR is a di-synaptic pathway with neurons of the cochlear root nucleus projecting directly to the facial nucleus without involving the ventral pontine reticular nucleus, which is the hub of the acoustic startle reflex (Hackley, 2015; Lee et al., 1996). The cochlear root nucleus is subject to PPI (Gomez-Nieto et al., 2010) and it is possible that modulation of the PAMR occurs at the level of the cochlear root nucleus rather than the pontine reticular nucleus where most PPI is thought to occur (Lingenhohl and Friauf, 1994). It would be useful to check whether the enlarged PAMR produced by activation of the neck and facial muscles also failed to produce any increase in the strength of GPIAS. Another factor that affects the size of the PAMR is the ear of stimulation, with contralateral acoustic stimulation sometimes producing a PAMR that is two or three times the size of the response produced by ipsilateral stimulation (Dus and Wilson, 1975) and binaural stimulation producing an even bigger response (Doubell et al., 2018). The effect of unilateral compared to bilateral stimulation should also be quantified with respect to GPIAS.

A potential limitation in the present study is that we only showed a clear eye-blink response in about 28% (2/7) of our participants and this is much lower than previous studies (Fournier and Hebert, 2013; Shadwick and Sun, 2014). This was presumably due to the small number of repeats but might also have been because we did not optimise the recording conditions for the eye-blink response (Blumenthal et al., 2005). Having the eye gaze to the right may have adversely interfered with the eye-blink response, and keeping the lights on in the recording booth may have increased the background activity in the orbicularis oculi muscle thus potentially partially masking the response. In addition the use of a monaural stimulus may have reduced the amplitude of the eye-blink response as binaural stimuli are usually used.

## 5 Conclusion

In conclusion, the similarity between the PAMR and the ear-flick response shown in rodents means that it may be possible to use the human PAMR to validate the GPIAS technique that has been used to detect tinnitus in guinea pigs (Berger et al., 2013; Coomber et al., 2014). The present results show that the PAMR is subject to GPIAS using similar parameters of gap and background noise to those used in rodents and with the human eye-blink (Fournier and Hebert, 2013; Shadwick and Sun, 2014; Turner et al., 2006). We are currently measuring the PAMR response in participants with tinnitus to determine if there are significant differences from an age-matched population when GPIAS is measured.
